# Low Radiation Dose Calcium Scoring: Evidence and Techniques

**DOI:** 10.1007/s12410-016-9373-1

**Published:** 2016-03-02

**Authors:** Kaitlin B. Baron, Andrew D. Choi, Marcus Y. Chen

**Affiliations:** Division of Cardiology, The George Washington University School of Medicine, Washington, DC USA; Advanced Cardiovascular Imaging Laboratory, Cardiovascular and Pulmonary Branch, National Heart, Lung and Blood Institute, National Institutes of Health, 10 Center Drive, Building 10, B1D416, Bethesda, MD 20891-1061 USA

**Keywords:** Coronary artery calcium (CAC) scoring, Low dose radiation, Iterative Reconstruction, Rescan variability, Coronary artery disease, Computed tomography (CT), Atherosclerotic cardiovascular disease (ASCVD)

## Abstract

Coronary computed tomography (CT) allows for the acquisition of thin slices of the heart and coronary arteries, which can be used to detect and quantify coronary artery calcium (CAC), a marker of atherosclerotic cardiovascular disease. Despite the proven clinical value in cardiac risk prognostication, there remain concerns regarding radiation exposure from CAC CT scans. There have been several recent technical advancements that allow for significant radiation dose reduction in CAC scoring. This paper reviews the clinical utility and recent literature in low radiation dose CAC scoring.

## Introduction

Coronary computed tomography (CT) allows for the acquisition of thin slices of the heart and coronary arteries, which can be used to detect and quantify coronary calcium, a marker of atherosclerotic cardiovascular disease (ASCVD). Given the utility of coronary artery calcium (CAC) scoring in assessing ASCVD risk, the 2010 American College of Cardiology Foundation/American Heart Association (“ACC/AHA”) Guideline for Assessment of Cardiovascular Risk in Asymptomatic Adults gave CAC scoring a class IIA recommendation suggesting that it was reasonable to consider in asymptomatic adults at intermediate coronary heart disease risk (10 to 20 % 10-year Framingham risk) [1]. However, the use of a CAC CT exposes the patient to ionizing radiation. A study from Kim KP, et al. has found significant variability in the radiation doses delivered at different sites performing CAC scoring with radiation doses ranging from 1 to even as high as 8–10 mSv [2]. Thus, the benefits of coronary artery calcium quantification should be weighted against the risks of exposure to ionizing radiation [1].

## The Clinical Use of Coronary Artery Calcium

The aforementioned 2010 ACC/AHA risk assessment guideline, which included six studies of 27,622 asymptomatic patients, found that the 11,815 patients that had CAC scores = 0 had a very low rate (0.4 %) of coronary heart disease deaths or myocardial infarction events over the subsequent 3 to 5 years [1]. A more recent paper by Valenti V, et al. has found that this “warranty period” for asymptomatic individuals without CAC extends out to 15 years [3•]. However, for those patients with a CAC score between 100 and 400, a CAC score of 400 to 1000, and a CAC score greater than 1000 were 4.3, 7.2, and 10.8 times more likely respectively to have an event [1]. In addition, in a landmark study from the Multi-Ethnic Society of Atherosclerosis, Detrano et al. analyzed data on 6722 patients in four racial or ethnic groups and found that increased CAC scores between 101 and 300 and above 300 were related to higher, almost 8-fold and almost 10-fold, respectively, increased risk of adverse ASCVD events regardless of baseline risk [4].

Thus, the general utility of CAC scoring has been in risk prognostication; that is, if patients are found to have an elevated CAC score, this portends a higher risk of ASCVD, which can be used to implement more aggressive CAD risk factor modification including aspirin and statin use as well as lifestyle modifications and increased adherence to medications [1]. In fact, Taylor et al. evaluated the association between CAC score and subsequent aspirin and statin usage in 1640 patients between ages 40 to 50 years in the Prospective Army Coronary Calcium Project cohort with a follow-up of six years and found that patients with a CAC score of greater than zero were three times more likely to receive a statin and also significantly more likely to receive an aspirin or both [5]. The EISNER study, a prospective randomized trial, assigned 2137 volunteers to either undergo or not undergo CAC scanning prior to risk factor counseling and found that those patients that received CAC scanning were more likely to have improved risk factor modification including improvement in systolic and diastolic blood pressure, total cholesterol, low-density lipoprotein cholesterol, triglycerides, weight, and Framingham risk score [6]. The authors also found that there was no increase in downstream medical testing or cost in the CAC scanning group because the increased resource utilization and cost in the subjects with CAC ≥ 400 was balanced by the decreased resource utilization and cost in the subjects with CAC = 0.

Subsequent to the 2010 ACC/AHA Guidelines on cardiovascular risk in asymptomatic patients, the 2013 ACC/AHA guidelines in cholesterol management recommendation to assess a patient’s ASCVD 10-year risk using the pooled cohort equations has increased the number of patients eligible for statin therapy [7]. After the release of these guidelines, Nasir et al. recently evaluated the implications of CAC scoring in reclassifying patients from a risk category in which statins are recommended to one in which they are not in the Multi-Ethnic Study of Atherosclerosis (MESA), which included 4758 patients between the ages of 45 and 75 with a median follow-up of about 10 years [8•]. Using the pooled cohort equations, 50 % of patients in the study were recommended for moderate- to high-intensity statins (for LDL ≥ 190, for LDL 70 to 189 in diabetics, or most commonly for 10-year risk ≥ 7.5 %), and 12 % were considered for moderate-intensity statins (ASCVD 10-year risk 5–7.5 %). Of those patients recommended for statins, 41 % had a CAC = 0 with a 5.2 ASCVD event rate/1000 person-years, and of those patients considered for moderate-intensity statins, 57 % had a CAC = 0 with a lower ASCVD event rate of 1.5/1000 person-years. Of those 38 % of patients who were not candidates for statin using the pooled cohort equations (ASCVD 10-year risk <5 %), a larger 79 % of patients had a CAC = 0 with an even lower ASCVD event rate of 1.2/1000 person-years. Overall, the absence of CAC reclassifies about 50 % of candidates as not eligible for statin therapy and results in a very low risk of future ASCVD events in asymptomatic adults.

While CAC scoring may further refine the risk categorization of patients, radiation exposure was one of the cited reasons that the updated 2013 ACC/AHA Guideline on the Assessment of Cardiovascular Risk downgraded CAC scoring from a class IIA recommendation in the 2010 ACC/AHA guidelines to a class IIB recommendation in asymptomatic, intermediate risk patients for whom the decision to treat based on risk is uncertain after formal risk estimation [9]. However, recent advances in radiation dose reduction for CAC scoring may enable a re-examination of these concerns.

## Strategies for Low Radiation Dose Coronary Artery Calcium Scoring

There are multiple methods to modulate radiation dose to follow the principles of “as low as reasonably achievable” or "ALARA” in CAC scoring. However, it is important to maintain the image quality within an acceptable degree of image noise. The Society of Cardiovascular Computed Tomography has published guidelines for several of these methods [10]. A brief summary of these guidelines follows.

First, acquiring the CT images via ECG-triggered axial or sequential imaging is important. It is advantageous to avoid retrospective ECG-gating. Secondly, current guidelines recommend CAC imaging at a peak tube voltage of 120 kVp. This threshold was chosen to maintain similar quantification to prior electron beam computed tomography (EBCT) [11]. This allows the use of 130 Hounsfield units (HU) as the standard threshold for quantifying CAC according the Agatston method. Low tube current values (in mA) are recommended and may be adjusted based on the patient’s body habitus. The scan length should be minimized to be limited to the coronary vasculature and the heart. This may be planned from the scout images. Slice thickness has been standardized to 2.5–3 mm based on the prior experience with EBCT [12]. With these standards in mind, there have been several recent advances in dose reduction strategies.

## Low kV/mA Calcium Scoring

Decreasing the tube current/voltage is a previously attempted method of reducing the radiation dose that has been evaluated in several studies; however, this methodology is found to cause a significant increase in image noise. Dey et al. evaluated 66 consecutive patients using an anthropomorphic heart/thorax phantom on a dual source CT (DSCT) (Siemens Medical Systems, Erlangen, Germany) at both standard 150 mAs and 85 or 120 mAs based on the patient’s body mass index and found no significant difference between Agatston and calcium volume scores when comparing individual data points [13]. There was 98 % agreement in Agatston score severity between the standard and low-dose scans. However, image noise was significantly higher for the low-dose scans (18.8 ± 5.5 HU) than that for standard scans (15.2 ± 4.8 HU), though both were within the target limits in guidelines.

To represent the same physical density at of CAC at a 130-HU threshold, Nakazato, et al. tested the effect of differing HU thresholds with tube current output using an anthropomorphic phantom and established that a 147-HU threshold at 100 kV would best correlate with a 130-HU threshold at 120 kV [14]. Subsequently, Marwan et al. evaluated 150 consecutive patients using high-pitch spiral acquisition on a DSCT (Siemens Medical Systems, Erlangen, Germany) at 120 kV (with a 130-HU threshold) and 100 kV (with the previously established 147-HU threshold and at a 130-HU threshold) and also found that mean image noise was significantly increased in the 100-kV radiation scans (130-HU threshold) compared to the standard radiation scans at 27 ± 7 and 20 ± 5 HU, respectively [15]. While there was overestimation at 100 kV (130-HU threshold), this improved with the 100 kV (147-HU threshold). With regard to zero scores, when compared to 120-kV imaging, three patients at 100 kV (147 HU) were reported to have calcium. Newton et al. utilized a 320-detector row CT (Toshiba Medical Systems, Tokyo, Japan) at tube currents from 40 to 300 mA adjusted to several body size parameters in 43 patients and demonstrated that using a scout attenuation coefficient allowed for the lowest potential reduction in mean radiation dose from 1.86 to 0.88 mSv while maintaining statistically significant correlation coefficients of 0.66 to 0.86 [16].

## Use of Iterative Reconstruction

**Fig. 1:**
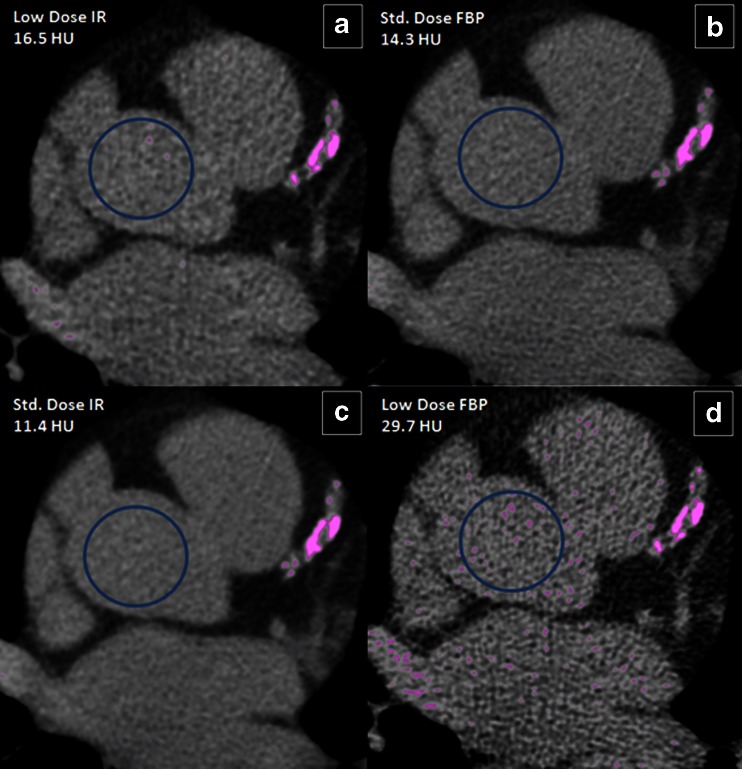
Example of CTs with filtered back projection (FBP) versus iterative reconstruction (IR) at standard radiation dose (“Std.”) and low radiation dose (“Low”). There is a *circle* signifying the region of interest (ROI) in the ascending aorta measuring the image noise as the standard deviation of the ROI in Hounsfield units (HU). Image noise is the highest for low dose FBP, while image noise between Std. Dose FBP and Low Dose IR is similar as evidenced by the HU measured by the ROI and the degree of pink speckling identified by the CAC scoring software. **a** Low Dose IR; **b** Std. Dose FBP; **c** Std. Dose IR; **d** Low Dose FBP

Iterative Reconstruction (IR) is a newer reconstruction algorithm that is being evaluated for use in CAC scoring as it allows for reduction in image noise, improved image quality, and lower radiation requirements (Fig. 1). IR methods have three steps: a forward projection of the volumetric object estimate creates artificial raw data (may be an image from the initial FBP), the real measured data is compared to the artificial raw data and creates a correction term, and the correction term is projected back onto the volumetric object estimate [17]. There are several IR techniques, which include algebraic reconstruction methods that model the geometry of the acquisition process, statistical methods that incorporate counting statistics of the detected photons, and model-based methods that model the acquisition process as accurately as possible. Research-based IR usually utilizes a combination of statistical and model-based IR techniques.

Iterative reconstruction has been evaluated in several domains, most notably in coronary CTA [18–20], as well as for other organ scanning such as lung and in re-operative cardiac surgery [21–23]. There have been several recent publications evaluating iterative reconstruction for CAC both in anthropomorphic phantoms and in patients at standard radiation doses with varying success (Table 1).

**Table 1:** Summary of selected studies evaluating the use of Iterative Reconstruction for Coronary Artery Calcium Scoring

Author	Year	*n*	IR algorithm	Vendor	Standard vs Low Radiation Median Agatston Scores
Gebhard, et al.[26]	2012	50	ASIR	GE Healthcare	837.3 (FBP) vs 709.2 (ASIR 100 %)
Kurata, et al.[25]	2013	70	SAFIRE	Siemens Healthcare	163.3 (FBP) vs 84.1 (SAFIRE 50 %)
Schindler, et al.[24•]	2014	110	IRIS/SAFIRE	Siemens Healthcare	76.0 (FBP) vs 75.7 (SAFIRE)
Van Osch, et al.[27]	2014	112	ASIR	GE Healthcare	81 (FBP) vs 53 (ASIR 100 %)
Obmann, et al.[29]	2015	68	HIR	Philips Healthcare	621.4 (FBP) vs 531.8 (L7) (CAC > 400)
Takahashi, et al.[28]	2015	352	ASIR	GE Healthcare	119 (FBP) vs 79 (ASIR 100 %)
Szilveszter, et al.[30]	2015	567	HIR/IMR	Philips Healthcare	147.7 (FBP) vs 107.0 (HIR) vs 115.1 (IMR)

*n* number of patients, *IR* iterative reconstruction, *IRIS* iterative reconstruction in image space, *SAFIRE* sinogram-affirmed iterative reconstruction, *AIDR3D* adaptive iterative dose reduction 3D, *ASIR* adaptive statistical iterative reconstruction, *HIR* hybrid iterative reconstruction, *IMR* iterative model reconstruction, *FBP* filtered back projection

Schindler et al. evaluated the influence of image-based and raw data-based IR algorithms on the Agatston score and subsequent cardiac risk stratification in 110 patients undergoing routine CAC scoring [24•]. A 64-DSCT scanner (Siemens Medical Systems, Erlangen, Germany) was used to obtain the phantom and patient images, which were reconstructed using traditional FBP, image-based iterative reconstruction (IRIS), and raw data-based sinogram-affirmed iterative reconstruction (SAFIRE). In vitro, mean Agatston scores for FBP, IRIS, and SAFIRE were comparable, and in the patient cohort, the Agatston scores were not significantly different for FBP, IRIS, and SAFIRE in paired comparisons. There was excellent agreement of categorization in Agatston risk percentiles with both IRIS and SAFIRE compared to FBP with identical percentile categories in 98.2 % with IRIS and 97.3 % with SAFIRE. The authors did notice that there was a non-significant trend toward lower scores for high-density lesions in the in vitro study using IR.

Kurata et al. also evaluated the effect of SAFIRE at 10, 20, 30, 40, and 50 % algorithms compared to FBP on CAC scoring in 70 patients using a 64-DSCT (Siemens Healthcare) [25]. An increased proportion of SAFIRE was significantly associated with a decrease in the CAC Agatston, volume, and mass scores, though attenuation within the aorta was unaffected. The 50 % SAFIRE can result in a negative CAC score while 10 % SAFIRE can increase the CAC score in a proportion of patients. There were three patients that had an Agatston score greater than zero using FBP that decreased to zero using more than 20 % SAFIRE.

Gebhard et al. studied the effect of applied adaptive statistical iterative reconstruction (ASIR) compared with FBP on CAC scoring in 50 patients within 90 days using a 64-slice CT scanner (GE Healthcare, Milwaukee, WI) [26]. The authors found that the use of ASIR algorithms at 20, 40, 60, 80, and 100 % was associated with a linear reduction in noise (median reduction of 50 %) and improvement in signal to noise ratio, but a significant decrease in CAC score. With increased percentage of ASIR, volume and Agatston scores (22 %) decreased. The authors postulated that the mass score might have lower susceptibility to partial volume effects. At 80 and 100 % ASIR, 18 % of patients were re-assigned to a lower-risk group while at 20 and 40 % ASIR, there was the least variation in Agatston score and significant noise reduction.

Van Osch et al. also evaluated the impact of ASIR on CAC scoring compared to FBP in 112 patients using a hybrid 64-slice single-photon emission CT/CT (SPECT/CT) (GE Healthcare, Milwaukee, WI) [27]. The authors only utilized a 100 % ASIR algorithm compared to FBP and found that Agatston, mass, and volume scores using ASIR were lower for all patients compared to FBP with a very strong correlation between Agatston scores using ASIR and FBP. Importantly, a large number of patients, 29 %, were moved to a lower-risk category using ASIR instead of FBP using five risk categories and 13 % of patients that had their Agatston score reduced to zero from greater than zero with FBP.

Takahashi et al. compared CAC scores in 352 consecutive patients using FBP (ASIR 0 %), ASIR-FBP composites (ASIR 30, 50, 70 %), and ASIR 100 % using a 64-slice multidetector CT (MDCT; GE Healthcare, Milwaukee, WI) and found that Agatston and calcium volume scores decreased as the percentage of ASIR increased and differed significantly among the five techniques [28]. Severity classification did not differ significantly between FBP and ASIR 30 % (Agatston score reduced by about 10 %), but did differ significantly between FBP and ASIR 50 %, FBP and ASIR 70 %, and FBP and ASIR 100 % with Agatston score reductions of about 17, 23, and 31 %, respectively. Noise decreased as the percentage of ASIR increased and among the three noise groups, the Agatston score was not significantly influenced by ASIR percentage.

Newer generation IR algorithms have been tested recently as well. Obmann et al. compared hybrid iterative reconstruction (HIR) to FBP in 68 patients using a 256-slice MDCT (Philips Healthcare, Cleveland, OH) and also found an excellent correlation between Agatston scores measured in all seven iteration levels with HIR and FBP [29]. In about 93 % of HIR reconstructions at all iteration levels, the patient assignment to a risk group was identical to that of the FBP reconstructions. Szilveszter et al. also compared the effect of HIR to FBP on CAC scoring in addition to iterative model-based reconstruction (IMR) using a 256-slice CT (Philips Healthcare, Cleveland, OH) in two different cohorts of patients: 63 symptomatic patients referred due to suspected CAD and 504 asymptomatic individuals in a test population from a National Health Examination survey [30]. The relative differences in median CAC scores were 7.2 % for HIR and 7.3 % for IMR in the patient population. There was statistical significance in CAC scores with HIR and IMR compared to FBP, but not between HIR and IMR. Using HIR and IMR, noise was reduced by 33.9 and 65.8 %, respectively, compared to FBP. In the test population, extrapolation of relative differences by IR algorithms yielded a 2.4 % change in risk stratification, but did not differ significantly among the three reconstructions and those 12 patients moved to lower-risk groups.

## Use of Low Dose Radiation with Iterative Reconstruction

After validation of IR algorithms with demonstrated improvements in image noise without change in risk stratification, several groups have tested the use of iterative reconstruction at low radiation dose. A brief summary of these studies follows (Table 2).

**Table 2:** Summary of selected studies evaluating low-dose radiation with Iterative Reconstruction for Coronary Artery Calcium Scoring

Author	Year	*n*	IR algorithm	Vendor	Standard vs low radiation effective dose
Hecht, et al.[31•]	2014	102	HIR	Philips Healthcare	0.76 vs 0.37 mSv (mean)
Matsuura, et al.[35]	2015	77	HIR	Philips Healthcare	1.20 vs 0.24 mSv (mean)
Willemink, et al.^(34^•^)^	2015	30	HIR	Philips Healthcare	0.7 vs 0.2 mSv (<80 kg pts only) (median)
Choi, et al.[32•]	2015	200	AIDR3D	Toshiba Medical Systems	1.38 vs 0.37 mSv (median)

*n* number of patients, *IR* iterative reconstruction, *HIR* hybrid iterative reconstruction, *AIDR* adaptive iterative dose reduction

Hecht et al. evaluated CAC scoring in 102 patients using a hybrid IR algorithm on a 256-slice CT scanner (Philips Healthcare, Cleveland, OH) at both standard radiation dosing and 50 % of the standard radiation dosing (with increased IR from seven to three) using a weight-based radiation dose algorithm [31•]. All patients were scanned using 120 kVp. The authors found that the correlation of the Agatston scores between the low and high dose was excellent (*r* = 0.998) with similar agreement for volume and mass scores. There were significant differences in the Agatston scores of 248.4 ± 497.1 vs. 237.9 ± 489.5 for standard vs. low radiation dose; however, this had little clinical significance. All of the patients in the lowest-risk category (zero CAC) and high-risk category (>400 CAC) remained in the same group at both doses, and 87 % of patients were in exact agreement in the low-to-intermediate CAC risk groups.

Choi AD et al. has demonstrated a significant reduction in radiation dose while maintaining excellent correlation in Agatston risk categories through the application of a novel adaptive iterative dose reduction 3D (AIDR3D) algorithm to CAC CTs [32•]. The authors evaluated 200 consecutive patients using 320-detector row CT (Toshiba Medical Systems, Otawara, Japan) and were able to achieve a 70 % reduction in radiation dose (1.38 mSv in standard dose vs. 0.37 mSv in low dose) while obtaining a 92 % agreement in Agatston risk category between standard and low dose radiation scans. The radiation dose was individually determined in an automated fashion by the CT scanner based on soft tissue attenuation of the scout images.

In looking at lowered radiation dose protocols in a cross-vendor fashion, Willemink et al. examined 15 ex vivo hearts with four different radiation protocols (4.1, 3.0, 1.9, and 0.8 mGy) using four different CT scanner vendors and found that lowering the radiation dose did not significantly change the Agatston, mass, or volume CAC scores [33•]. Another study by Willemink et al. evaluated the maximum achievable dose reduction with IR using an anthropomorphic calcium scoring phantom and a subsequent within-patient study on a 256-slice CT (Philips Healthcare, Cleveland, OH) in 30 patients that each received the four CT scans in a single session at 100, 60, 40, and 20 % of the reference radiation dose with FBP and HIR levels 1, 4, and 7 [34]. In the patient population, median Agatston scores increased with FBP while they decreased with IR at 20 % of the reference dose. Volume and mass scores decreased with increasing levels of IR. The authors were able to achieve an 80 % reduction in radiation dose with reclassification rate within 15 % if the highest level of IR is applied. In the phantom study, Agatston scores remained unchanged between 55 and 20 mAs (40 % of the approximate reference dose).

Matsuura et al. examined the lowest tube current and highest iDose level for CAC scoring in 77 consecutive patients using a 256-slice MDCT (Philips Healthcare, Cleveland, OH) [35]. Each patient had two non-contrast CT scans at normal tube current 364 mA (80 and 16 mAs) for FBP and low current 73 mA (16 mAs) with iDose level 7. The percentage difference between FBP and HIR for the Agatston, volume, and mass scores were 20.7, 20.7, and 27.1 %, respectively. Between FBP at standard tube current and HIR at low tube current, there was no systematic bias in the three different scores using the Bland-Altman analysis.

## Rescan Variability

Coupled with the need for radiation dose reduction, it is important to recognize that interscan variability exists in CAC. Even while utilizing low radiation dose strategies, the ability to achieve low interscan variability is critically important for CAC scoring given its prognostic significance for coronary artery disease risk stratification and subsequent medical management. Interscan variability can be affected by the type of scanner, the type of reconstruction algorithm, the heart rate, and the density of the calcification. Several studies have compared various CT scanners and reconstruction algorithms to evaluate the reproducibility of CAC scoring.

Ghadri et al. examined the interscan variability of CAC scoring on a 64-slice single source multi-detector CT (GE Healthcare, Milwaukee, WI) and a 64-slice dual source CT (DSCT; Siemens Medical Systems, Erlangen, Germany) in thirty patients that were scanned on both CT scanners within 23 ± 27 days (range 0–90 days) [36]. There was an excellent interscan agreement of Agatston scores (*r* = 0.976) with a coefficient of variation of 15.1 %. The interscan agreement was best for Agatston scores of <1000 and decreased in patients that had extensive calcifications. Mass scores and volume scores also demonstrated excellent correlation (*r* = 0.975 and *r* = 0.971, respectively), though volume scores had higher coefficient of variability of 44.9 %. The authors felt that the variability in volume scores was more likely related to the different software systems rather than the different scanner types.

Detrano et al. evaluated the effect of two different types of CT scanners and the type of calcium measurement on the interscan variability of CAC scoring from the previously cited Multi-Ethnic Study of Atherosclerosis (MESA) cohort of 6741 patients [37]. Each patient had two scans for CAC scoring on either an electron-beam CT or a MDCT. The authors also found excellent agreement between EBCT and MDCT scans of about 96 % (*k* = 0.92), but with overall mean relative rescan differences of 20.1 % for Agatston score and 18.3 % for calcium volume and interpolated volume scores. EBCTs were more likely to demonstrate noise artifacts, and MDCTs were more likely to demonstrate motion and misregistration artifacts.

The aforementioned study by Willemink et al. examined the interscan variability of four different CT vendors and IR algorithms (Philips Healthcare with iDose levels 1 and 6, Toshiba Medical Systems with AIDR mild and strong, GE Healthcare with ASIR 20 and 60 %, and Siemens Healthcare with SAFIRE 1 and 5) on CAC scoring in five ex vivo cadaveric hearts [33•]. The authors found significant differences in Agatston scores between the four vendors with median Agatston scores ranging from 332 to 469. Notably, scans done using the CT scanner/software from GE Healthcare resulted in the highest Agatston scores and the lowest calcification and mass scores.

Lastly, in the study by Choi AD, et al., the authors also evaluated the interscan reproducibility of standard and low radiation dose scans in 200 patients that were scanned twice at a standard radiation dose and twice at a low radiation dose on a 320-detector row MDCT (Toshiba Medical Systems, Otawara, Japan) [32•]. There was an excellent rescan agreement of Agatston CAC scoring classification with low IR (91 %, *k* = 0.87), standard FBP (93 %, *k* = 0.91), standard IR (92 %, *k* = 0.89), and low FBP (90 %, *k* = 0.88).

## Conclusions

It is important to recognize that the design of appropriate studies that advance the field of low radiation dose calcium scoring may raise important ethical implications of radiation exposure to patients. In the most recent studies cited this paper, the combined radiation doses of multiple scans generally fell within an accepted community standard for radiation exposure. However, it is reasonable to ask at what threshold the added radiation dose of the repeated scans would be justified when weighed against the risks of the exposure. These risks are balanced by the potential large-scale benefit that would be provided to patients through practice implantation of these techniques. The underlying principles fall within the principles outlined by the Declaration of Helsinki [38]. It is reasonable to expect that these studies are vetted carefully by institutional review boards and that only in the absence of alternative study designs should this type of research be pursued [39].

In conclusion, we now have an increasing body of evidence on multiple platforms that CAC scoring at sub-mSv radiation doses can be performed reliably, particularly through the use of iterative reconstruction. Future studies include newer generation IR algorithms, combining IR algorithms with low-kV scanning as well as newer technologies such as dual energy scanning that may permit even lower-energy scanning. Thus, the present literature supports coronary calcium scoring at radiation doses that allow for cardiac risk categorization lower than for screening in other disease states such as mammography (0.7 mSv) [40], lung cancer (1–2 mSv) [41], or colon cancer (4–5 msV) [42].
